# Errors in Abstract, Table, and Discussion

**DOI:** 10.1001/jamanetworkopen.2021.2581

**Published:** 2021-03-01

In the Original Investigation titled “Effect of Bimagrumab vs Placebo on Body Fat Mass Among Adults With Type 2 Diabetes and Obesity: A Phase 2 Randomized Clinical Trial,”^1^ published January 13, 2021, there were errors in the Abstract, Table, and Discussion. In the Abstract, the change in hemoglobin A_1c_ in the placebo group should have been 0.04 percentage points. In Table 2, the *P* value for the Matsuda index should have been .10. The first paragraph of the Discussion should have referred to a 0.5% decrease in body fat mass and a 0.04–percentage increase in hemoglobin A_1c_ in the group that received placebo and lifestyle intervention. This article has been corrected.^1^
